# *Ralstonia mannitolilytica*: an emerging multidrug-resistant opportunistic pathogen in a tertiary care hospital setting

**DOI:** 10.1099/acmi.0.000367

**Published:** 2022-05-31

**Authors:** Tasneem Siddiqui, Sangram Singh Patel, Richa Sinha, Ujjala Ghoshal, Chinmoy Sahu

**Affiliations:** ^1^​ Department of Microbiology, Sanjay Gandhi Post Graduate Institute of Medical Sciences, Lucknow, India

**Keywords:** *Ralstonia mannitolilytica*, nosocomial pathogen, Gram-negative, MALDI-TOF-MS

## Abstract

**Introduction.:**

*
Ralstonia mannitolilytica* is a rare opportunistic pathogen capable of causing a serious infection in immunocompromised patients. Our objective was to describe all cases of *R. mannitolilytica* bloodstream infection identified within 2 years at our tertiary care centre, focusing on clinical characteristics, risk factors, antibiotic sensitivity patterns, management and outcomes.

**Case Series.:**

We compiled a descriptive case series including 14 non-duplicate *R. mannitolilytica* isolates obtained from bloodstream infection samples from the microbiology laboratory of a tertiary care centre from June 2019 to June 2021. All isolates were initially identified based on their morphological properties and biochemical reactions, and then underwent matrix-assisted laser desorption/ionization time-of-flight mass spectrometry (MALDI-TOF-MS) examination for confirmation of identity. Antibiotic susceptibility testing was performed using the Kirby–Bauer disc diffusion method and Vitek 2. All 14 patients presented with symptoms of fever and/or chills, and a positive blood culture for *R. mannitolilytica *. After 48 h of incubation, no *Ralstonia* growth was reported from any of the current environmental or pharmaceutical water samples. Chemotherapy (9/14), mechanical ventilation (4/14), steroid use (2/14) and diabetes mellitus (1/14) were associated risk factors in our patients. The antibiotic sensitivity panel showed maximum resistance to aminoglycosides (64.3%) and no resistance to cefoperazone/sulbactum. Patients received treatment with cefoperazone/sulbactum and meropenem or ceftazidime. Thirteen patients recovered with antibiotic therapy and one patient succumbed to his illness.

**Conclusion.:**

*
R. mannitolilytica* can cause bloodstream infections in immunocompromised patients. It is likely to be missed or underreported due to lack of clinical awareness. MALDI-TOF MS is helpful in rapid identification. *R. mannitolilytica* is resistant to many routinely used antibiotics, including carbapenems.

## Introduction

*Ralstonia mannitolilytica* – aerobic Gram-negative non-fermentative rods – are environmental organisms commonly found in water and soil, but are now emerging as opportunistic pathogens causing infections in immunocompromised patients [1]. They could be an aetiological agent in common source nosocomial outbreaks due to contamination of parenteral fluid and medical equipment that is considered to be sterile [2]. *R. mannitolilytica* are known to cause sepsis, meningitis and central venous catheter-associated bacteraemia [3]. Of late several case reports of infections by *R. mannitolilytica* have been seen from India and worldwide [4–12]. Many case reports have highlighted *R. mannitolilytica* as causing bacteraemia and sepsis [4–11]. Lampropoulos *et al.* (2021) and Rajendran *et al.* (2022) highlighted the organism as an emerging opportunistic pathogen causing sepsis in neonatal intensive care units [9, 10] . Carreira *et al.* (2020) emphasized the role of *R. mannitolilytica* in endocarditis [12]. The tendency of *R.mannitolilytica* to form biofilm enhances the organism’s survival in the environment (including the hospital environment), and plays a role in frequent antibiotic resistance [13].

There are currently no clear treatment guidelines or Clinical and Laboratory Standards Institute (CLSI) breakpoints for *R. mannitolilytica *. Treatment is challenging, as this species is frequently resistant to many antibiotics [1]. Resistance to many of the β-lactam class of antibiotics, including the carbapenems, is generally observed in *R. mannitolilytica *. blaOXA-22 and blaOXA-60 are class D carabapenmase genes that are commonly associated with *Ralstonia* species [14]. Treatment is based on the antibiotic susceptibility profile of the isolate [1]. There are very few case reports on this rare opportunistic pathogen in bloodstream infections from India. In this case series, we summarize the clinical characteristics of 14 patients with nosocomial bloodstream infections caused by *R.mannitolilytica* in the last 2 years, and analyse their risk factors, management and outcomes. We have also reported drug susceptibility patterns, which may help in the management of patients infected with this bacterium.

## Case series

This case series includes clinically significant non-duplicate culture isolates of *Ralstonia* species obtained from suspected bloodstream infections in microbiology laboratory of a tertiary care centre of northern India. These cases occurred between June 2019 and June 2021 in the Departments of Haematology, Critical Care medicine, Gastro-medicine, Pulmonary Medicine and Endocrine Surgery. Written informed consent for publication of clinical details was obtained from the patients before the study. A study proforma was designed, which included patient demographic data, clinical details, underlying risk factors during the episode of infection, duration of hospital stay, treatment received and outcomes.

### Sample processing and identification of isolates

Blood samples were inoculated into aerobic and anaerobic blood culture bottles (Becton Dickinson Diagnostics, USA) and incubated in the in BACTEC blood culture system (BD Diagnostics, USA). Once flagged positive, a Gram stain was performed from the bottle and the broth was plated onto 5 % sheep blood agar and MacConkey agar. The plates were incubated at 37 °C in ambient air and then inspected for growth at 24 h and again at 48 h. All of the culture media used were obtained from HiMedia Laboratories (Mumbai, India). The bacterial isolates were first identified using the routine staining and biochemical tests used in our laboratory [15]. The biochemical reactions for this Gram-negative bacillus revealed the results as follows: catalase-positive, oxidase-positive, motile, non-fermenting, methyl red-negative, Voges–Proskauer-negative, indole-negative, triple sugar iron agar – K/K (alkaline/alkaline), and urease-negative. Citrate was utilized but aesculin and gelatin were not hydrolyzed. *R. mannitolilytica* and *Ralstonia pickettii* were differentiated on the basis of nitrate reduction (negative in *R. mannitolilytica *) and acidification of d-arabitol and mannitol (both negative in *R. pickettii *) [16]. The identity of all the isolates was confirmed by matrix-assisted laser desorption/ionization time-of-flight mass spectrometry (MALDI-TOF MS) using the VITEK MS system (bioMérieux, Marcy-l’Etoile, France). Briefly, one bacterial colony of each isolate was spotted directly onto a single well of a disposable, barcode-labelled Vitek MS-DS target slide (bioMérieux, Marcy l’Etoile, France) and overlaid with 1 µl of saturated α-cyano-4-hydroxycinnamic acid (CHCA) (Vitek MS-CHCA, bioMérieux, Marcy l’Etoile, France) matrix and then air-dried. *Escherichia coli* ATCC 8739 was used as the quality control strain and was transferred directly to designated spots on the target slide as per the recommendation of the manufacturer. The target slide with all prepared isolates was then loaded into the VITEK MS system to acquire the mass spectra of bacterial proteins [17]. Finally, the mass spectra acquired for each isolate were compared to the known mass spectra contained in the SARAMIS database. The software compares the spectra and generates a numerical value (score value) based on the similarities between the observed and stored data sets. A score value above 2.0 is generally considered to be a valid species level identification and values between 2.0 and 1.7 represent reliable genus-level identification.

### Antimicrobial susceptibility testing

Antimicrobial susceptibility testing was performed using the Kirby–Bauer disc diffusion method on Müller–Hinton agar and with an automated method (Vitek 2, bioMérieux) [18]. Antibiotic sensitivity was tested as per the Clinical and Laboratory Standards Institute (CLSI) guidelines [19]. As there were no CLSI breakpoints or zone diameters available for *R. mannitolilytica *, the results were interpreted using the CLSI breakpoints for *Pseudomonas* spp. [19]. *P. aeruginosa* ATCC 27853 and *E. coli* ATCC 25922 were put up as controls.

### Environmental surveillance sampling

Environmental samples were collected from different wards from which *R. mannitolilytica* isolates had been obtained by an infection control nurse. Commercially available sterile swabs (HiMedia Laboratories, Mumbai, India) were used to collect samples from the patients’ immediate surroundings, bed rails, tubing and medical devices. Samples from unused sterile intravenous fluids, liquid soaps and disinfectants and water (drinking water and tap water) were obtained in a sterile universal container. Air sampling was performed using a sieve impactor.

### Processing of environmental samples

Swabs were incubated in brain heart infusion (BHI) media at 37 °C for 18–24 h. After 24 h, BHI media was inspected visually for any turbidity or growth. A small volume of sample was then taken with the help of an inoculating loop and sub-cultured on blood and MacConkey agar. The culture plates were further incubated for 24 h at 37 °C. Any positive growth was further identified using Gram staining and appropriate biochemical tests.

### Statistical analysis

Statistical tests were performed using SPSS for Windows version 14 (SPSS, Inc., Chicago, IL, USA) for descriptive statistics. Categorical data were described using numbers and percentages.

## Results

A total of 14 cases of *R. mannitolilytica* were reported from our hospital over a period of 2 years. All of the patients presented with symptoms of fever and/or chills, and a positive blood culture for *R. mannitolilytica *. We carried out comprehensive environmental sampling from various sites in our hospital. However, after 48 h of incubation all of the cultures were found to be sterile. The age of the patient population ranged from 8 to 58 years with a median age of 15.5 years. The number of males and females enrolled in the study were 10 and 4, respectively, with a M : F ratio of 2.5 :  1. Detailed demographic and clinical characteristics of the patients are illustrated in Table 1. The majority of the patients with *R. mannitolilytica* infection had haematological malignancy (8/14) and there was one patient each with breast carcinoma, dengue haemorrhagic shock syndrome, decompensated chronic liver disease, acute respiratory distress syndrome, acute necrotizing pancreatitis and severe coronavirus disease 2019 (COVID-19) pneumonia. The average duration of hospital stay was 29 days (range 10–58 days) and the mean time of development of infection after hospitalization was 15.1 days. Chemotherapy (9/14), mechanical ventilation (4/14) and steroid (2/14) use were the most common risk factors in these patients.

**Table 1.** Demographic and clinical characteristics of patients

Patient no.	Age range	Clinical diagnosis	Risk factors
1	21–30	Dengue haemorrhagic shock syndrome	Gastrointestinal surgery within 30 days, mechanical ventilation, steroid use
2	51–60	Decompensated chronic liver disease	Diabetes mellitus
3	51–60	Acute respiratory distress syndrome	Mechanical ventilation, anti-tubercular treatment
4	31–40	Acute necrotizing pancreatitis	Mechanical ventilation
5	11–20	Acute lymphoblastic leukaemia	Chemotherapy
6	51–60	Severe COVID-19 pneumonia	Mechanical ventilation, steroids
7	11–20	Acute myeloid leukaemia	Chemotherapy
8	51–60	Breast carcinoma	Chemotherapy
9	11–20	Acute myeloid leukaemia	Chemotherapy
10	1–10	Hodgkin’s lymphoma	Chemotherapy
11	11–20	Hodgkin’s lymphoma	Chemotherapy
12	11–20	Acute myeloid leukaemia	Chemotherapy
13	11–20	Acute lymphoblastic leukaemia	Chemotherapy
14	11–20	Acute myeloid leukaemia	Chemotherapy

**Fig. 1.**
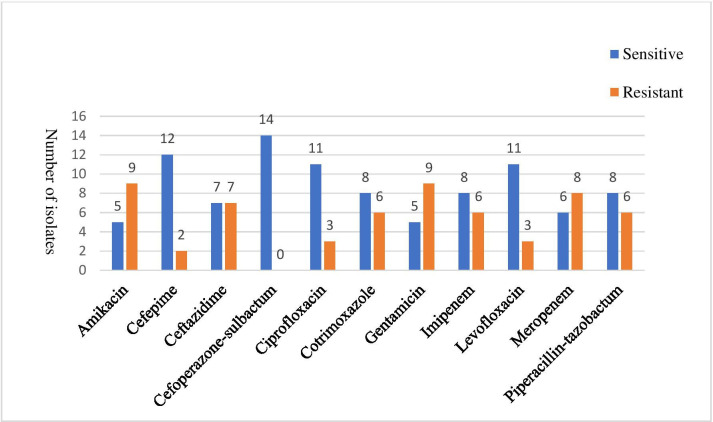
Antimicrobial susceptibility of 14 clinical isolates of *R. mannitolilytica* determined using the CLSI standards and interpreted as per the CLSI breakpoints for *Pseudomonas* spp.

MALDI-TOF-MS identified all 14 isolates as *R. mannitolilytica* with confidence values of 99.9 %. All of the isolates (100 %) were sensitive to cefoperazone/sulbactum (Fig. 1). It was determined that 64.3 % (9/14) and 50 % (7/14) of isolates were resistant to aminoglycosides (amikacin and gentamicin) and ceftazidime, respectively; 42.8 % (6/14) of isolates were resistant to each of piperacillin/tazobactam, cotrimoxazole and carbapenems (imipenem and meropenem); and 21.4 % (3/14) and 14.3 % (2/14) of isolates were resistant to fluoroquinolones (ciprofloxacin and levofloxacin) and cefepime, respectively.

The following antibiotics were administered to patients after antibiotic susceptibility testing and according to the clinicians’ decisions: cefoperazone/sulbactam was given to 10 (71.4 %) patients, meropenem to 3 (21.4 %) patients and ceftazidime to 1 (7.2 %) patient. Thirteen patients recovered with antibiotic therapy and one patient succumbed to his illness, but the cause of death for this patient was complications of COVID-19 (Table 2).

**Table 2.** Timeline of infection, identification, management and outcome for the patients

Patient no.	Duration of hospital stay (days)	Time from admission to development of infection (days)	Identity of isolates (MALDI-TOF)	Treatment received	Outcome/follow-up
1	11	5	* R. mannitolilytica *	Cefoperazone/sulbactum	Recovered
2	15	11	* R. mannitolilytica *	Cefoperazone/sulbactum	Recovered
3	58	27	* R. mannitolilytica *	Cefoperazone/sulbactum	Recovered
4	35	5	* R. mannitolilytica *	Cefoperazone/sulbactum	Recovered
5	10	8	* R. mannitolilytica *	Meropenem+colistin	Recovered
6	49	26	* R. mannitolilytica *	Meropenem+colistin	Dead
7	20	8	* R. mannitolilytica *	Cefoperazone/sulbactum	Recovered
8	10	6	* R. mannitolilytica *	Meropenem+colistin	Recovered
9	21	6	* R. mannitolilytica *	Cefoperazone/sulbactum	Recovered
10	40	30	* R. mannitolilytica *	Cefoperazone/sulbactum	Recovered
11	29	25	* R. mannitolilytica *	Cefoperazone/sulbactum	Recovered
12	52	32	* R. mannitolilytica *	Ceftazidime+amikacin	Recovered
13	31	12	* R. mannitolilytica *	Cefoperazone/sulbactum	Recovered
14	28	10	* R. mannitolilytica *	Cefoperazone/sulbactum	Recovered

## Discussion and conclusion

Few cases of infections caused by *R.mannitolilytica* have been reported due to limited awareness of the pathogen. The development of modern medical care, inappropriate and unnecessary use of broad-spectrum antibiotics, and the extensive use of various immunosuppressants have caused increased rates of opportunistic infections from organisms such as *R.mannitolilytica*. The prevalence of *Ralstonia* infection is increasing notably, even without person-to-person transmission [1]. *Ralstonia* exist widely in external aqueous environments, including municipal water and medical water purification systems [20, 21]. As the bacteria can pass through 0.2 µm filters during the sterilization process, medical products may be contaminated during the manufacturing phase [22]. *Ralstonia* can create biofilms on the surfaces of medical supplies and produce toxins [13]. Most infectious cases caused by *Ralstonia* species are due to the use of contaminated solutions, chlorhexidine, saline solution, blood products and sterile water as well as the colonization of medical devices (tap water and water used for haemodialysis, bronchoscope flushing and heparin for flushing) [3, 23, 24].

In this case series we have described 14 nosocomial cases of bloodstream infections caused by *R. mannitolilytica *, although we could not track their source. All of our patients were at high risk of infection. The high-risk factors listed in the literature are cancer, blood vessel catheters, mechanical ventilation and other immunocompromised conditions [1, 25] that were also found in our study. The most frequent risk factor in the study population was the use of chemotherapy and steroids, which could be why most of the patients in the study had malignancy, which itself is an immunocompromised state [3]. Further, these patients received chemotherapy as well as immunosuppressant drugs [3]. Hence, such patients are vulnerable to opportunistic infections such as *Ralstonia *, which is non-harmful to healthy persons [1]. Long-term use of steroids also causes an increase in the rate of infections due to deranged cellular immunity. Central lines are essential in malignancy patients for long-term chemotherapy infusions, and infection of central lines might happen during the insertion procedure as well as during the maintenance period. Biofilm formation in the central lines might cause bacteria to harbour and cause central line-associated bloodstream infections [4]. Blood products through central lines might help in the formation of biofilms that might cause central line infections in these cases. It is well accepted that, over time, the presence of endotracheal tubes for mechanical ventilation increases the risk of bacterial colonization and the development of infection. Similarly, it is conceivable that *Ralstonia *, a water-borne bacterium cultured from respiratory devices, may have the capacity to colonize the airways; its capacity to generate biofilms, like *Pseudomonas* species, enables it to cause infection in patients who are mechanically ventilated for longer periods [21]. Diabetes itself causes increased infections due to unbalanced blood sugar levels in patients. All of these patients had prolonged hospital stays, which might have led to acquisition of this bacterium. Boattini *et al.* (2018) reported that this pathogen is an important cause of nosocomial bacteraemia in diabetics, preterm infants, solid organ and haematological malignancy patients, and patients with end-stage renal disease [4]. Thomas *et al.* (2021) reported it to cause bacteraemia and gastroenteritis in a patient with rheumatoid arthritis.

Diagnosis and management of *Ralstonia* spp. infections is challenging. First, there is the difficulty of correctly identifying and differentiating between *Ralstonia* spp. members using routine laboratory analyses, because they have very similar biochemical patterns to each other and to other bacterial genera, such as the *Burkholderia cepacia* complex [1]. MALDI-TOF showed good performance regarding the identification of *R. mannitolilytica* in a previous study using isolates from patients with cystic fibrosis [26]. Although 16S rDNA is the reference method for identifying micro-organisms, it is costly and cumbersome. In the present study, MALDI-TOF correctly identified all of the isolates rapidly. Therefore, in comparison to the turnaround time and PCR identification cost of the 16S sequencing method, MALDI-TOF MS would be a better choice for identification of *R. mannitolilytica* . Our results support the general consensus that MALDI-TOF MS can provide rapid and accurate results [27]. Second, *R. mannitolilytica* are frequently resistant to numerous different types of antibiotics, including several beta-lactams and most of the aminoglycosides [1]. The organism may produce various enzymes that can hydrolyze antibiotics. These can confer resistance to a broad range of antibiotics, including benzylpenicillin, narrow-spectrum cephalosporins, ceftazidime, aztreonam and the carbapenems [26]. As currently there are no clear treatment guidelines for *R. mannitolilytica* and the data from various case reports reveal a notable heterogeneity in the percentage of antibiotic resistance amongst *R. mannitolilytica* isolates, in the course of treatment we advocate using antibiotic susceptibility testing to adjust the use of antimicrobial agents. Our antibiotic susceptibility pattern showed maximum resistance to aminoglycosides and no resistance to cefoperazone/sulbactum. Hence most of our patients were treated with cefoperazone/sulbactum or a with some other third-generation cephalosporins. Daxboeck *et al*. reported carbapenem resistance in 12 out of their 30 strains [28], which is consistent with our study, where we found carbapenem resistance in 6 out of 14 isolates. Thus meropenem was only used for seriously ill patients whose antibiograms showed sensitivity to meropenem. A 93 % (13/14) recovery rate in the study patients supports the view that early diagnosis and early initiation of appropriate antibiotics are required for good outcomes in these patients.

Even though *R. mannitolilytica* is not recognized as a major pathogen, clinicians and microbiologists should pay attention to the potential of this opportunistic bacterium, which is able to cause bloodstream infections, as it has certain notable characteristics, such as multidrug resistance, the ability to survive in water supplies and resistance to disinfection practices. Prompt diagnosis and subsequent administration of antibiotics in line with antimicrobial susceptibility testing results are needed to clear infections. MALDI-TOF MS is helpful for rapid identification and *R. mannitolilytica* is capable of being resistant to many routinely used antibiotics, including carbapenems.
